# Effects of Styrene-Acrylic Sizing on the Mechanical Properties of Carbon Fiber Thermoplastic Towpregs and Their Composites

**DOI:** 10.3390/molecules23030547

**Published:** 2018-03-01

**Authors:** Sean Bowman, Qiuran Jiang, Hafeezullah Memon, Yiping Qiu, Wanshuang Liu, Yi Wei

**Affiliations:** 1 Donghua University Center for Civil Aviation Composites, Donghua University, 2999 North Renmin Road, Shanghai 201620, China; sean.bowman@hotmail.fr (S.B.); hafeezullah_m@yahoo.com (H.M.); 2 Key Laboratory of Textile Science & Technology, Ministry of Education, College of Textiles, Donghua University, 2999 North Renmin Road, Shanghai 201620, China; jj@dhu.edu.cn (Q.J.); ypqiu@dhu.edu.cn (Y.Q.)

**Keywords:** carbon-fiber tows, styrene-acrylic sizing, towpregs, carbon fiber-thermoplastic composites

## Abstract

Thermoplastic towpregs are convenient and scalable raw materials for the fabrication of continuous fiber-reinforced thermoplastic matrix composites. In this paper, the potential to employ epoxy and styrene-acrylic sizing agents was evaluated for the making of carbon fiber thermoplastic towpregs via a powder-coating method. The protective effects and thermal stability of these sizing agents were investigated by single fiber tensile test and differential scanning calorimetry (DSC) measurement. The results indicate that the epoxy sizing agent provides better protection to carbon fibers, but it cannot be used for thermoplastic towpreg processing due to its poor chemical stability at high temperature. The bending rigidity of the tows and towpregs with two styrene-acrylic sizing agents was measured by cantilever and Kawabata methods. The styrene-acrylic sized towpregs show low torque values, and are suitable for further processing, such as weaving, preforming, and winding. Finally, composite panels were fabricated directly from the towpregs by hot compression molding. Both of the composite panels show superior flexural strength (>400 MPa), flexural modulus (>63 GPa), and interlaminar shear strength (>27 MPa), indicating the applicability of these two styrene-acrylic sizing agents for carbon fiber thermoplastic towpregs.

## 1. Introduction

Carbon-fiber-reinforced polymer composites (CFRPs) have been increasingly used during the past decades due to their potential for significant weight saving [1,2,3]. Historically, thermoset resins have been used as matrix for CFRPs [4,5,6], but the requirements for thermoplastic matrix have been elevated during the past decade [7,8,9]. Compared with thermosets, thermoplastics are inherently tough [10,11,12], recyclable, and have an unlimited shelf life [13]. However, CFRPs are more difficult to manufacture using thermoplastic matrix due to its poor processibility [14]. Currently, several methods, such as pultrusion and hot compression molding, have been developed to fabricate CFRPs with thermoplastic matrix [15]. Among them, the use of thermoplastic towpregs is one convenient way to fabricate CFRPs [16]. So far, thermoplastic towpregs can be made by different processing technologies, such as wet powder impregnation or dry powder impregnation. The powder-coating process draws the tow through a cloud of resin particles then heats up to partially wet and consolidate it [17].

Besides polymer matrix, carbon fibers play a major role in the mechanical performances of final CFRPs [18,19,20]. However, due to the brittle nature of carbon fibers, the carbon-fiber tows are inherently easy to generate fuzz for during processing and thus require protection [21]. In general, the surfaces of carbon fibers are protected by coating them with a thin layer of sizing, which can also improve the wettability as well as processibility of carbon fibers [22,23,24]. The most common type of sizing agent for carbon fibers is based on epoxy [25,26]. This type of sizing agent can be well-compatible with thermoset matrix, but it is usually incompatible with thermoplastic matrix because the chemical and interaction mechanisms taking place at the interphase are different [27,28]. Therefore, thermoplastic or polymeric sizing agents are highly desired for meeting the increasing demand for CFRPs using thermoplastic matrix. So far, many thermoplastic polymers, such as polyamide, polyether sulfone, and polyetheretherketon, have been used as sizing agents for carbon fibers [22,29,30,31,32]. The type of sizing agent to be used depends on the characteristics of the thermoplastic matrix. Among the thermoplastic sizing agents, styrene-acrylic sizing has an excellent combination of good adhesion to many thermoplastic matrices, UV resistance, tensile/elongation balance, and low cost [33,34,35].

In this work, different types of sizing agents for carbon fibers were studied to verify their compatibility for the fabrication of thermoplastic towpregs via a powder-coating process. Styrene-acrylic sizing agents with different formulations were compared with the traditional epoxy sizing agent in terms of protection and thermal stability. Polyamide 6 (PA6) is used as the matrix resin to prepare the towpregs due to its good chemical resistance, processibility, mechanical properties, and low cost compared to other polyamides [36]. The influences of styrene-acrylic sizing agents on the bending rigidity of carbon-fiber tows and towpregs were investigated by the cantilever and Kawabata methods. The towpregs with different styrene-acrylic sizing agents were used to fabricate composite panels by hot compression molding. The mechanical properties of the carbon-fiber composites, including their flexural and interlaminar shear strength, were investigated.

## 2. Results and Discussion

### 2.1. Thermal Stability and Sizing Content

**Figure 1:**
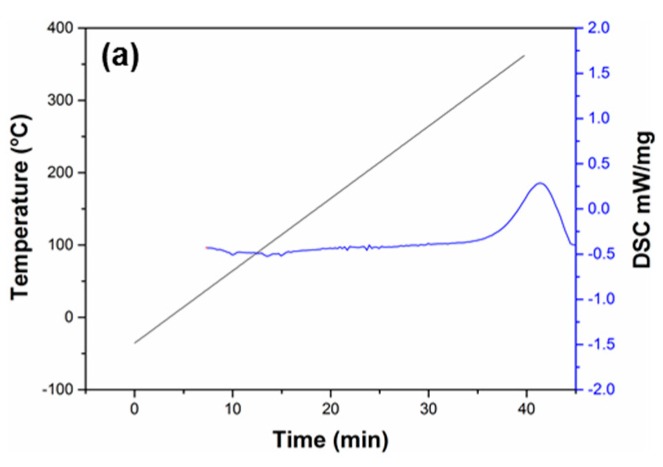

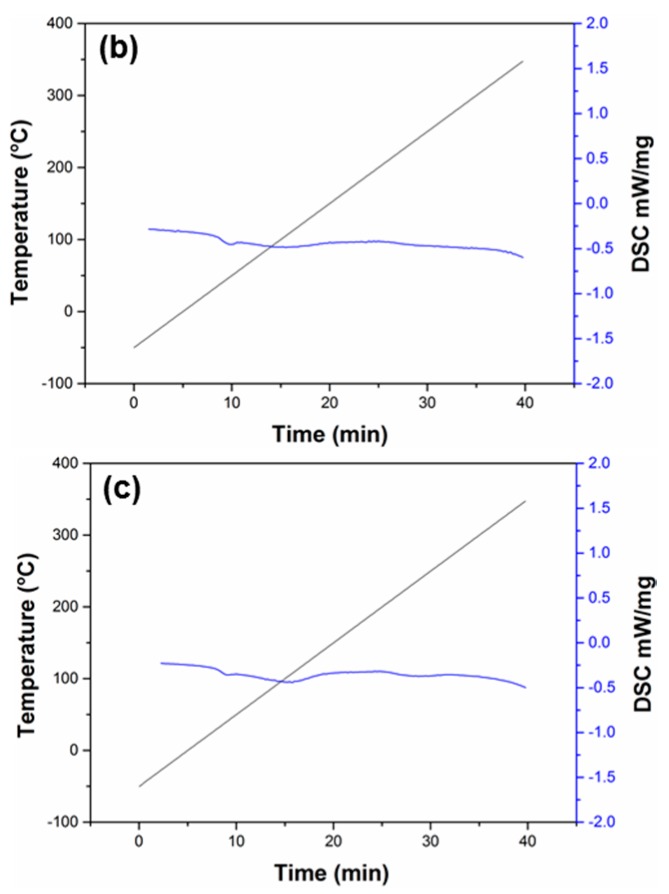
DSC plots of the carbon fibers with S-EP (**a**), S-SA1 (**b**), and S-SA2 (**c**) sizing agents.

**Figure 2:**
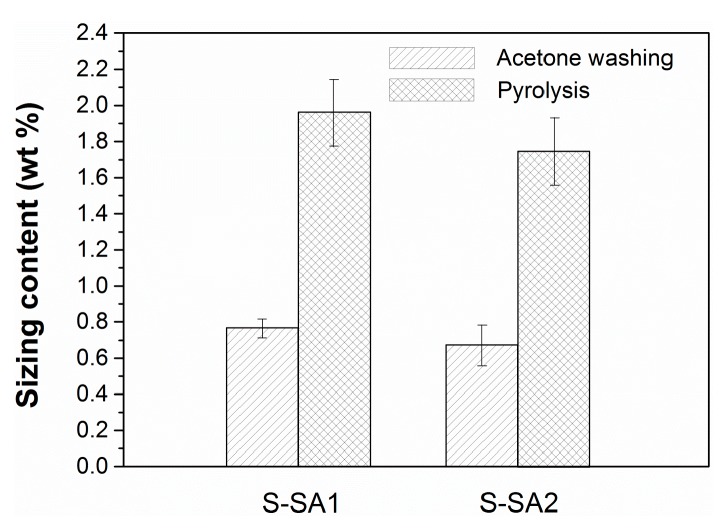
Sizing contents of the carbon fibers with S-SA1 and S-SA2 sizing agents measured by the acetone washing and pyrolysis methods.

Good thermal stability is highly desired for the powder-coating method to prepare towpregs because of the high processing temperature. Thermal degradation was not observed for all of the sizing materials in the test temperature range. However, a clear exothermic peak appears above ~300 °C for the epoxy sizing agent as shown in Figure 1a, indicating the reactions of epoxy groups. The poor thermal stability may be the reason why towpregs cannot be produced from carbon-fiber tows with epoxy sizing agent due to the plugging of the processing line. In contrast, the two styrene-acrylic sizing agents remain relatively stable up to 350 °C as shown in Figure 1b,c. The sizing contents of carbon fibers with styrene-acrylic sizing were measured by the acetone washing and pyrolysis methods (Figure 2). The results obtained by the pyrolysis method show that the average sizing contents of carbon fibers with S-AS3a and S-AS3b are 1.96 and 1.74 wt %, respectively. The average sizing contents of carbon fibers with S-AS3a and S-AS3b tested by the acetone-washing method are 0.77 and 0.67 wt %, respectively. These results indicate that the styrene-acrylic sizing agents cannot be fully removed even after repeated washing in a Soxhlet apparatus with acetone. The pyrolysis method would be more reliable.

### 2.2. Protective Effects of Different Sizing Agents

**Figure 3:**
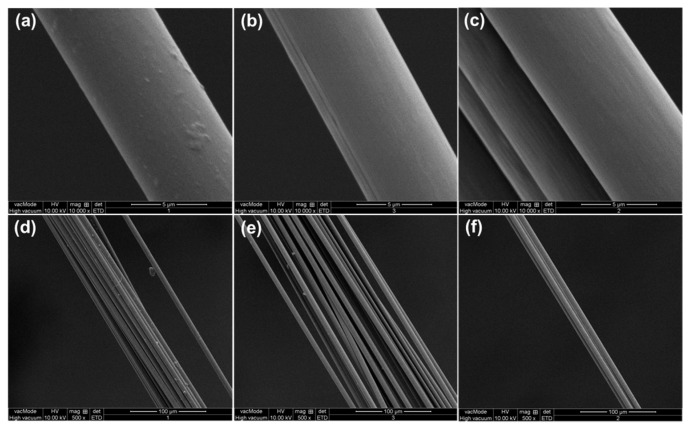
SEM images of the carbon fibers with S-EP (**a**,**d**); S-SA1 (**b**,**e**); and S-SA2 (**c**,**f**) sizing agents. The scale bars in **a**–**c** and **d**–**f** are 5 and 100 µm, respectively.

**Figure 4:**
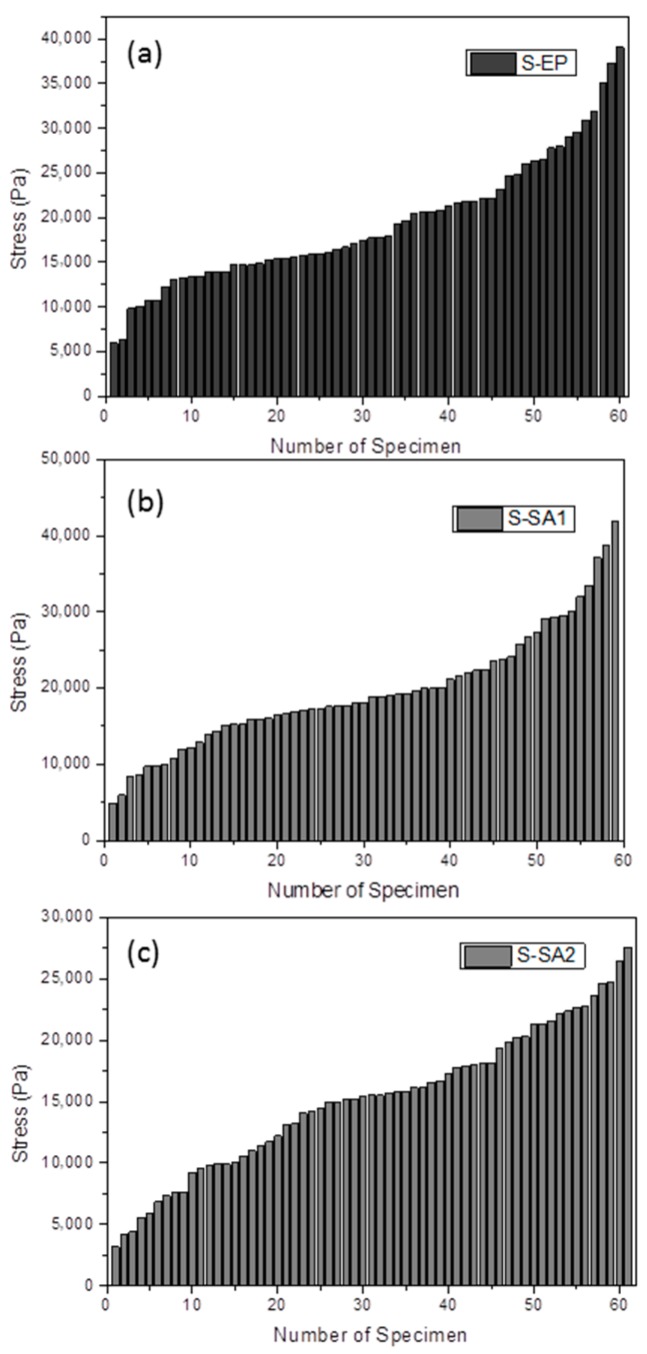
Failure Stress of the carbon fibers with S-EP (**a**); S-SA1 (**b**); and S-SA2 (**c**) sizing agents measured by single fiber tensile tests.

The surface morphologies of the sized carbon fibers were observed by SEM (Figure 3). As can be seen, the surfaces of carbon fibers with styrene-acrylic sizing are quite smooth, indicating that the sizing agents were uniformly coated. In contrast, some bumps can be observed on the surface of carbon fibers with epoxy sizing. The protective effects of different sizing agents were investigated by single fiber tensile test, and the results are shown in Figure 4. The dimensions tested and methods used are described in the sections above. The data processing was inspired by the survival probability deduced from Weibull’s two-parameter model [37,38]. This model is based on a weakest link theory, in which the failure is considered to come from the weakest part of the fibers. For a carbon fiber with length *L*, it can be divided into a number of equal parts “*n*”, and each part may be “strong” or “weak”. The carbon fibers which are not well-protected by the sizing agents would have more weak parts compared with those are well-protected. According to the two-parameter Weibull distribution, the probability of failure (*P_f_*) can be calculated according to Equation (1). The derivation of this equation can be found in detail elsewhere [37].

(1) $$P_{f}=1-\exp\left(-L{\left(\frac{\sigma }{\sigma_{o}}\right)}^{m}\right)$$

where *L* is the length of the fiber, *σ* is the failure stress, *σ_o_* is the shape factor, and *m* is the scale factor. It should be noted that in this study a full determination of all of these parameters is not done. The real stress failures were computed for comparison but without measuring the parameters themselves. The *P_f_* values are in inverse proportion to the failure stresses for the carbon fibers at a given diameter and length. Herein, the failure stresses of carbon fibers are categorized into four levels: *P*_1_, *P*_2_, *P*_3_, and *P*_4_, which represent the failure stresses in the ranges of 0–20, 25–50, 50–75, and 75–100% of the maximum measured stress, respectively. The *P_n_* distribution can preliminarily reflect the protective effects and property consistency of the sized carbon fibers.

As shown in Table 1, the carbon fibers with S-SA1 sizing show a slightly higher maximum failure stress (41.9 MPa) and a similar average failure Stress (19.6 MPa) compared to the carbon fibers with S-EP sizing. The carbon fibers with S-SA2 sizing show the lowest maximum (27.6 MPa) and average (15.1 MPa) failure stresses. The consistency of failure stress for the carbon fibers sized by S-EP is superior to those sized by S-SA1, because 44% of specimens with S-EP sizing have failure stresses over 50% of the maximum value, while the percentage for the specimens with S-SA1 sizing is 33%. It should be noted that the carbon fibers with S-SA2 sizing show the best property consistency and 66% of specimens have failure stress over 50% of the maximum failure stress, although they have the lowest maximum and average failure stress.

### 2.3. Bending Rigidity of Carbon-Fiber Tows

**Figure 5:**
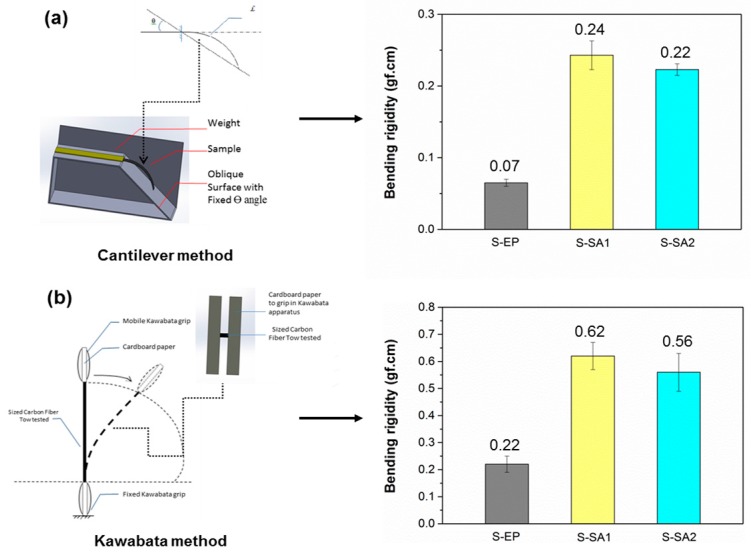
Schematic of the cantilever (**a**) and Kawabata (**b**) methods as well as the corresponding bending rigidity of the carbon-fiber tows with S-EP, S-SA1, and S-SA2 sizing agents.

It is known that sufficient stiffness of carbon-fiber tows is required to fabricate towpregs. The bending rigidity of carbon-fiber tows with epoxy and styrene-acrylic sizing agents were measured by the cantilever and Kawabata methods. As shown in Figure 5a,b, the bending rigidity values obtained from the two methods show some differences, which are due to the different constraint manners. The differences can be explained as follows: for the Kawabata test (Figure 5b), a smaller part of the tow is tested and the measurement is significantly affected by the inter-fiber interactions, such as internal friction between filaments. This method can provide a very precise measurement with a given curvature. In contrast, the filaments in the carbon-fiber tows might slip more easily when the cantilever method was used (Figure 5a), resulting in lower bending rigidity values. The tows with styrene-acrylic sizing showed higher bending rigidity compared to those with epoxy sizing. This is because the un-cured or partially cured epoxy sizing agent on the surfaces of carbon-fiber filaments is in the form of a sticky liquid film at room temperature, whereas the thermoplastic sizing agent is in the form of a solid film.

### 2.4. Evaluation of the Towpregs

**Figure 6:**
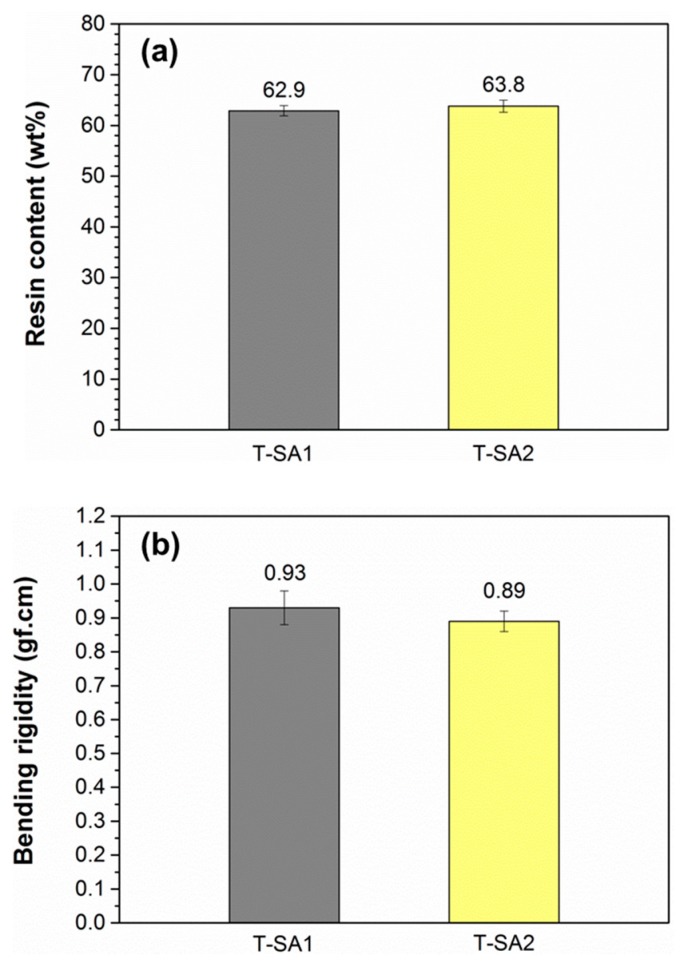
Resin content (**a**) and bending rigidity (**b**) of towpregs T-SA1 and T-AS2.

To investigate the flexibility of towpregs after coating them with PA6 resin matrix, the bending rigidity of towpregs was measured by the Kawabata method. Unlike carbon-fiber tows, the flexibility of towpregs is dominated by the amounts of coated PA6 matrix and less affected by the sizing agent. The resin contents of two towpregs were determined by the acid-digestion method. As shown in Figure 6, the towpregs T-SA1 and T-SA2 have similar average resin contents, which are 62.9 and 63.8%, respectively. The average bending rigidity values of T-SA1 and T-SA2 are 0.93 and 0.89 gf·cm, respectively, which are low enough for further processing, such as weaving, preforming, and winding [39,40].

### 2.5. Mechanical Performances of Composite Panel

**Figure 7:**
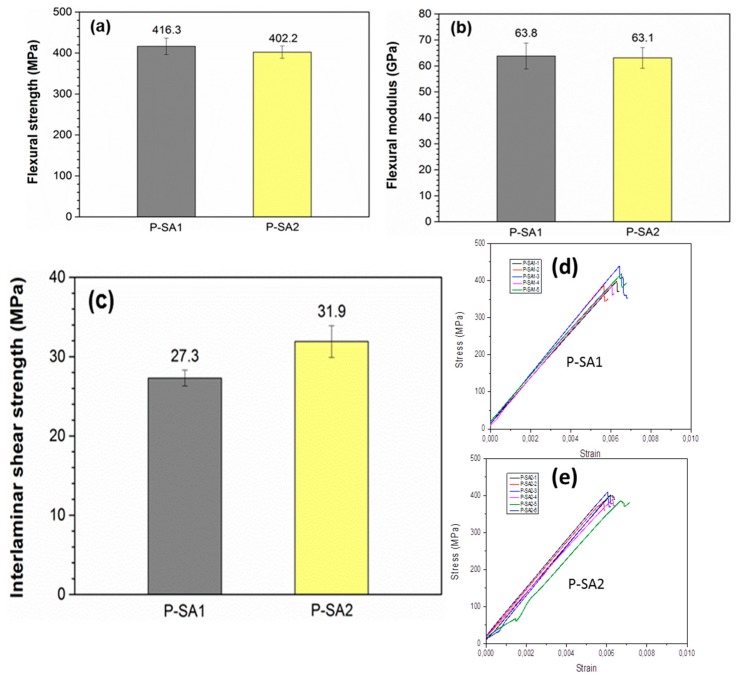
Flexural strength (**a**); flexural modulus (**b**); and interlaminar shear strength (**c**) of the P-SA1 and P-SA2 composite panels; (**d**) details of the three points flexure test for P-SA1; and (**e**) details of the three-point flexure test for P-SA2.

Composite panels P-SA1 and P-SA2 were fabricated from T-SA1 and T-SA2 via hot compression molding following the conditions and dimensions of samples specified earlier. Figure 7a,b show the flexural properties of P-SA1 and P-SA2. The *V*_f_ values obtained for both panels were 28.6% and 29.1%, respectively, with void contents lower than 1%. The flexural strength of P-SA1 (416.3 MPa) is slightly higher than that of P-SA2 (402.2 MPa), but the two composite panels have a similar flexural modulus, which is about 63–64 MPa. It is noteworthy that the flexural strength and modulus of the composite panels prepared by the towpregs using Primospire^TM^ PR-120 (a polymer developed by Solvay Advanced Polymers) and 760 Tex M30SC (Torayca) are 124.3 MPa and 30.0 GPa [41], respectively, which are much lower than the values of P-SA1 and P-SA2. To explain this result, the interfacial interaction between fiber and thermoplastic matrix was evaluated by short beam shear test [42]. Although interlaminar shear strength does not depend solely on fiber–resin bonding [43,44], the short beam shear test is still considered to be an effective method. As shown in Figure 7c, the interlaminar shear strengths of P-SA1 and P-SA2 are 27.3 and 31.9 MPa, respectively. The strengths are nearly twice the value (15.0 MPa) of the carbon fiber/PA6 composite reported by Ma et al. [45]. The additive in S-SA2 shows a clear impact on the interfacial interactions between the fiber tows and matrix resin. Compared to P-SA1, P-SA2 showed about a 17% increase in interlaminar shear strength. For the relationship between flexural strength and interlaminar shear strength, the following explanation may be made: due to its large molecular weight, this sizing is immobile on the surface of a carbon fiber when further processed during part fabrication; therefore, it may have acted as a linkage between the carbon fiber and the matrix resin. Its proper balance of polarity may have made it bond well to the carbon fiber’s surface and the matrix resin. The lower bonding, and hence lower interlaminar strength, may allow the fiber to stretch more during a flexural test, thus increasing the flexural strength (the valid failure mode in a flexural test is tensile failure).

## 3. Materials and Methods

### 3.1. Materials

SYT 45 (12K) carbon fibers were provided by Zhongfu Shenying Carbon Fiber Co. Ltd., Lianyungang, China. Specifications are show in Table 2. The conventional epoxy sizing agent (S-EP) and two styrene-acrylic (S-SA1 and S-SA2) sizing agents with proprietary formulations were provided by Beijing Eastern Acrylic Chemical Tech Co., Ltd., Beijing, China. The difference between S-SA1 and S-SA2 lies in the type of additives added to the formulations. Ultramid PA6 was used as thermoplastic matrix and was purchased from BASF Inc., Ludwigshafen, Germany. Sulfuric acid (H_2_SO_4_, 95–97%), acetone, and hydrogen peroxide (H_2_O_2_, 30%) were purchased from Sinopharm Chemical Reagent Co., Ltd., Shanghai, China.

### 3.2. Sizing Process and Fabrication of Towpregs and Composite Panels

The carbon fibers were sized by S-EP, S-SA1, and S-SA2 using the industrial production line of Zhongfu Shenying Carbon Fiber Co. Ltd. The process parameters were controlled closely and kept constant for all of the sizings studied. The sized tows were further processed into towpregs by Fibrtech Inc. (Atlanta, GA, USA) using Ultramid PA6 as matrix via a dry-powder method. The towpregs made from carbon-fiber tows with S-SA1 sizing and S-SA2 sizing are referred to as T-SA1 and T-SA2, respectively. The carbon-fiber tows with S-EP sizing cannot be made into towpregs because the epoxy sizing agent was not compatible with the high-temperature processing required for the dry-powder method. Composite panels were conveniently fabricated from the towpregs with a (0/90)5s configuration by hot compression molding (250 °C, 0.3 MPa, 15 min). The composite panels fabricated from T-SA1 and T-SA2 were named P-SA1 and P-SA2, respectively.

### 3.3. Characterization

The thermal stabilities of the three sizing agents were measured by differential scanning calorimetry (DSC) on a Netzsch 214 calorimeter. The temperature range was set from −50 to 350 °C at a heating rate of 10 °C min^−1^. The contents of the sizing agents were determined by the acetone washing and pyrolysis methods. The carbon-fiber tows were pre-dried in a vacuum oven at 85 °C for 24 h. For the pyrolysis method, the carbon-fiber tows were heated up to 600 °C for 2 h in a furnace under a Nitrogen atmosphere. For the washing method, carbon fibers were treated in a Soxhlet apparatus (Shanghai Heqi Glassware Co. Ltd, Shanghai, China) with acetone for 48 h. The obtained carbon-fiber tows were dried and weighed. The sizing contents were calculated according to the weight variation of the carbon-fiber tows.

The bending rigidities of the sized carbon-fiber tows and towpregs were measured by two methods. One approach was performed based on the cantilever test following ASTM D1388. The bending rigidity (*B*) can be calculated according to the following equation (Equation (2)).

(2) $$B=q\cdot D^{3}$$

where *q* is the weight per unit area and *D* is bending length. *D* was defined by Peirce as Equation (3)

(3) D=I·f(θ)

(4) f(θ)=12·(cosθ2tanθ)13

where *I* is the cross-sectional moment of inertia with the neutral axis perpendicular to the plane of bending, *f*(θ) is the geometric factor, and θ is the angle. *f*(θ) can be obtained by Equation (4).

The bending rigidity was also calculated from Equation (4) according to the Kawabata method as follows:

(5) $$K=0\;\;\;\;\;\;\;if\;M<M_{0}\;B.K=M-\mathrm{sign}(K).M_{0}\;\;\;\;\;if\;M\geq M_{0}$$

where *K* is the centerline curvature, *M* is the bending moment, and *M_o_* is a threshold accounting for different physical phenomena, such as frictional restraint.

The single fiber tensile test was used to evaluate the protective effects of different sizing agents. It was conducted on an Electronic Single Fiber Strength Test machine (Model 12PSB, Qinsun instrument Co., Shanghai, China). Single fibers from the sized tows were put between the clamps of the machine and tested in tension. More than fifty specimens for each type of sized carbon fiber were tested with an initial length of 10 mm, and the distribution of failure force was made based on the test results.

The resin content of the towpregs was measured using the acid digestion method (ASTM D3171). The dried and pre-weighed towpregs were immersed in H_2_SO_4_ (95–97%) and heated to 55 °C. Then, the blend was mixed with H_2_O_2_ (30%, V_H2SO4_:V_H2O2_ = 7:3) and digested for 60 min. After filtration, the obtained carbon fibers were washed with excess deionized water, dried in a vacuum oven (60 °C for 12 h), and weighed. The resin contents were calculated according to the weight loss of the towpregs.

The flexural properties of composite panels were measured according to ASTM D-790 on a universal testing machine MTS 810 (MTS Systems Ltd., Beijing, China). The dimension of each specimen was 140 × 15 × 2.5 mm and the speed of the crosshead was 2.0 mm min^−1^. The interlaminar shear strength was measured by short beam shear (SBS) tests following the ASTM-D2344 standard. The dimension of each specimen was 15 × 5 × 2.5 mm and the speed of the crosshead was 1.0 mm min^−1^. Each reported value was the average of at least five specimens. The average fiber volume fraction (*V*_f_) of the composite panels was obtained using the acid-washing method following ASTM-D3171 with similar digestion conditions as the towpregs. The *V*_f_ found was equivalent for each panel: around 28.6% and 29.1%. Scanning electron microscopy (SEM) was conducted using a Quanta 250 Scanning Electron Microscope (Thermo Fisher Scientific Inc., Hillsboro, OR, USA). The samples were coated with gold for 60 s.

## 4. Conclusions

In this work, we demonstrated the feasibility of fabricating thermoplastic towpregs using two styrene-acrylic sizing agents via a dry powder-coating method. DSC results indicate that the styrene-acrylic sizings have sufficient thermal stability for the high-temperature powder-coating process. The results of the single fiber tensile tests show that the styrene-acrylic sizing can offer good protection to carbon fibers, though the effectiveness of protection is slightly inferior compared to the traditional epoxy sizing. The results of bending rigidity tests suggest that the carbon-fiber tows with the two styrene-acrylic sizings increased stiffness compared to those with epoxy sizing. It was demonstrated that the prepared towpregs have sufficient flexibility for further processing, such as weaving, preforming, and winding. The results of the flexural and short beam shear tests indicate that the composite panels fabricated from the towpregs have good mechanical properties and fiber–matrix bonding strength. It is worth noting that this study was mainly limited to the macroscopic and mesoscopic level. The study of the interfacial interaction between sizing and carbon fibers at the molecular level will be reported in subsequent papers.

## Figures and Tables

**Table 1:** Failure stress and its distribution of the carbon fibers with different sizing agents.

Sized Fiber	S-SA1	S-SA2	S-EP
Max Stress (MPa)	41.9	27.6	39.1
Mean value (MPa)	19.6	15.1	19.3
*P*_1_ (%)	13	10	3
*P*_2_ (%)	54	26	53
*P*_3_ (%)	25	44	33
*P*_4_ (%)	8	20	11

**Table 2:** SYT 45 Specifications.

Tow	Spec	Tensile Strength (GPa)	Tensile Modulus (GPa)	Elongation (%)	Linear Density (g/km)	Density (g/cm^3^)	Diameter (μm)
**SYT 45 Carbon Fiber**	12k	4.5	240	1.9	800	1.80	7
